# Barriers to methamphetamine treatment seeking in residential centers in Aguascalientes, Mexico

**DOI:** 10.3389/fpsyg.2023.1193453

**Published:** 2023-08-21

**Authors:** Kalina Isela Martínez Martínez, Yancarlo Lizandro Ojeda Aguilar, Lorenia Robles Villarreal, María Abigail Paz Pérez

**Affiliations:** Department of Psychology, Autonomous University of Aguascalientes, Aguascalientes, Mexico

**Keywords:** barrieres, seeking treatment, residential centers, methamphetamine use, age of onset meth, machine learning

## Abstract

**Introduction:**

In the Aguascalientes, most people who seek treatment go to non-governmental residential centers, and about half request treatment for meth use. Although some barriers to treatment seeking among these users are known, few studies have been conducted with the Mexican population, specifically with users of residential centers. The aim of this study was to explore the main barriers reported by these patients, the relationship between reported barriers and meth use, as well as identify possible user profiles based on the barriers and the pattern of consumption.

**Methods:**

We designed a brief survey that evaluated sociodemographic data, consumption pattern, help-seeking for consumption and use of services, barriers in the search for services, depression, and suicide attempts. Here, we report the results of barriers and consumption patterns. The study sample consisted of 865 individuals receiving treatment for meth use in 23 certified residential centers.

**Results:**

Patients reported an average of 2.12 barriers, the main ones being not considering the services useful for them (41.6%), not considering it important to attend (35%), and not finding time to attend the consultation (29.8%). We found a statistically significant relationship, although weak, between the number of barriers reported by participants and the age of onset of meth use, dangerous perception of meth use, attempts to quit, and the number of problems associated with use. We used a cluster analysis that was performed using the k-means machine learning algorithm, which revealed two clusters. The first was formed by patients who started using meth at a young age which has more problems associated with meth use and more barriers in seeking services, while the other was formed by patients who started at an older age which have fewer problems and fewer barriers. We found statistical differences between groups, where it was found that young group reported consuming more substances, more problems associated, and more barriers in seeking services.

**Discussions:**

This study revealed the main barriers to seeking treatment among patients in residential centers and found that the age of onset of meth use is a risk factor for presenting more barriers and more problems associated with consumption.

## 1. Introduction

Substance consumption is a public health problem (National Institute on Drug Abuse [NIDA], 2017). Worldwide, 284 million people between the ages of 15 and 64 have used drugs at some point in their lives, and their use increased by more than 25% from 2010 to 2020 (United Nations Office on Drugs and Crime [UNODC], 2022). According to the UNODC, in its World Drug Report, the use of amphetamine-type stimulants (ATS) has increased significantly in recent years worldwide (United Nations Office on Drugs and Crime [UNODC], 2022). Methamphetamine or meth is a type of ATS, similar in structure to amphetamines, which is highly addictive, and affects the user’s nervous system (National Institute on Drug Abuse [NIDA], 2022). It is also the most consumed by people in treatment throughout the world (United Nations Office on Drugs and Crime [UNODC], 2022).

In Mexico, there are two main sources that offer treatment for substance use: governmental and non-governmental services. The former provides outpatient and residential treatment and consist of the Specialized Medical Units-Primary Addiction Care Centers (UNEME-CAPA for Spanish acronym) and Care Units of the Youth Integration Centers (CIJ for Spanish acronym; National Commission Against Addictions Comisión Nacional contra las Adicciones [CONADIC], 2020 for Spanish acronym).

On the other hand, non-governmental services, also known as residential centers or annexes, mostly provide residential care through mutual, mixed, and professional aid models and include 1,299 residential centers distributed in the 32 states of the Mexican Republic (Epidemiological Surveillance System for Addictions [SISVEA for Spanish acronym]; Sistema de Vigilancia Epidemiológica de las Adicciones [SISVEA], 2020). It is important to clarify that not all residential centers in Mexico are establishments certified by CONADIC, so not all of them report their activities to official sources and their relevant data is unknown. Residential centers reported that the impact drug for which they sought treatment was alcohol, followed by meth, marijuana, cocaine, inhalants, heroin, and tobacco. On the other hand, at the UNEME-CAPA, a total of 53,588 people sought treatment for issues related to substance use, with meth use showing a significant increase from 2014 to 2016, rising by 3.2%.

Comisión Nacional contra las Adicciones, responsible for collecting information from governmental and non-governmental residential centers, reported in its most recent data that in 2020; 101,142 people requested treatment for addictions in both types of services, from most of whom (30.2%; *n* = 30,545) sought help for some ATS as their impact drug (Comisión Nacional contra las Adicciones [CONADIC], 2020). Meanwhile, the SISEVA reported that in the same year, they provided care to 59,360 people, 58.6% of the population reported by CONADIC, of whom 47.5% (*n* = 28,185) sought treatment for meth use (Sistema de Vigilancia Epidemiológica de las Adicciones [SISVEA], 2020).

The Mexican state of Aguascalientes was above the national average in this comparison. Out of the 2,694 people who requested treatment in both types of services, 1,690 went to residential centers (62.7%) of whom 66.9% (*n* = 1,130) had meth as their impact drug (Comisión Nacional contra las Adicciones [CONADIC], 2020; Sistema de Vigilancia Epidemiológica de las Adicciones [SISVEA], 2020).

These results indicate that in Mexico, and in the state of Aguascalientes, most people seeking treatment go to residential centers, and about half of these individuals request treatment for meth use. In addition, there has been an increase in consumption among the youth and adolescent population, with the onset of use occurring at younger ages (Auerbach et al., 2018), making this issue more common.

According to the National Survey of Psychiatric Epidemiology, people with substance use disorders take an average of 10 years before seeking treatment (Borges et al., 2007) and only 17.7% of them use health services (Borges et al., 2006). Therefore, it is important to understand the barriers that this population faces when seeking treatment for their consumption.

Some authors have classified the barriers into two central axes: attitudinal and structural. The first is explained by treatment expectations and the stigma associated with seeking treatment; while the latter refers to economic factors, location of treatment, and the lack of information (Crisp et al., 2000; Issakidis and Andrews, 2002; Saldivia et al., 2004; Sareen et al., 2007; Benjet et al., 2020).

A meta-analysis conducted in 2016 aimed to report barriers to accessing services that provide treatment for meth use (Cumming et al., 2016). The meta-analysis included 11 studies from five countries. The authors identified four categories or types of barriers: (1) psychological or internal; (2) practical; (3) suitability of services; and (4) barriers from service providers, with psychological or internal barriers being the most prevalent in the majority of studies. The four psychological barriers were stigma, beliefs that treatment is unnecessary, preferring to be alone without assistance, and confidentiality and privacy concerns (Cumming et al., 2016).

Similar results have been found in other studies, in which the main barriers among women meth users in Australia are individual stigma, intrapersonal violence, and institutional stigma (Clifford et al., 2023). On the other hand, it has been reported that users who perceive their meth use as non-problematic face more barriers than those who perceive it as problematic (Quinn et al., 2013). Other studies have found that both internal barriers (low self-efficacy, conflicting thoughts about meth use, and withdrawal symptoms) and external barriers (escaping the drug environment, friends and family preventing recovery, and inadequate rehabilitation programs) may be factors that prevent recovery from meth use (Alexander et al., 2018).

Despite some known barriers to seeking treatment for meth users, few studies have been conducted on the Mexican population, particularly with users of residential centers. The objective of this study was to identify the main barriers reported by people seeking treatment for meth use in certified residential centers in the state of Aguascalientes, the relationship between reported barriers and meth use and identify possible user patterns based on the relationship between the variables.

## 2. Materials and methods

A cross-sectional exploratory study was conducted to identify the main barriers to seeking treatment and their relationship with meth use among patients receiving treatment in certified residential centers in the state of Aguascalientes, Mexico.

### 2.1. Participants

The participants were 865 patients receiving treatment for substance use at one of the 23 certified or in-process-of-certification residential centers by CONADIC. Convenience sampling was used to select the centers through the Mental Health Department of the Institute of Health Services of the State of Aguascalientes (ISSEA for Spanish acronym). The inclusion criteria were that patients voluntarily agreed to participate in the study and receive treatment specifically for meth use.

### 2.2. Instrument

We designed a brief survey (BS) consisting of 69 questions that evaluated 6 global variables: sociodemographic data (6), consumption history (10), seeking help with meth use and use of services (12), barriers to seeking services (1), depression (35) and suicide attempts (5). For the purposes of this study, we only report data related to the consumption history and barriers to seeking services. See Martínez et al. (2023) for a detailed description of these data.

The items that measured consumption history, and its numerical values, were as follows:

1. Total drugs used: We asked, “What substances have you consumed?” and presented a list of different substances for the participant to select. This variable represents the sum of each selected option.
2. Meth use onset age: We asked, “How old were you the first time you consumed meth?”
3. Time consuming (years): Calculated as the participant’s current age minus the age of onset.
4. Annual consumption: We asked, “How many times have you consumed meth in the last year?” The response options included “I did not consume it,” “1 to 3 times,” “3 to 11 times,” and “More than 12 times.” The values for monthly consumption options were: I did not consume it = 0, 1 to 3 times = 1, 4 to 11 times = 2, and More than 12 times = 3.
5. Monthly consumption: We asked, “How many times have you consumed meth in the last month?” The response options were like the previous question.
6. Dangerous perception of meth use: We asked, “How do you perceive your meth use?” with response options of “Not dangerous,” “Dangerous,” and “Very dangerous.” The values for this answer options were: It is not dangerous = 0, It is dangerous = 1, It is very dangerous = 2.
7. Longest period without meth use: We asked, “What has been the longest period during which you have not consumed meth?” The response options were “0 days,” “1 to 7 days,” “8 days to 1 month,” and “2 to 6 months.” Here, the values for answer options were: From 2 to 6 months = 0, From 8 days to 1 month = 1, From 1 to 7 days = 2, and 0 days = 3.
8. Attempts to stop consumption: We asked, “Have you tried to stop consuming meth?” with possible answers of “Yes” or “No.” Values for answer options were obtained with: No = 0, Yes = 1.
9. Desire to stop consuming: We asked, “At this time, would you like to decrease your meth use or stop completely?” with possible answers of “Yes” or “No.” The values for the response options were like the previous question.
10. Problems associated with consumption: We asked, “Have you experienced any of the following problems due to your meth use? (Select all that apply)” Participants could select multiple options from a provided list or add their own if not listed. This variable represents the sum of each selected option.

To measure the barriers associated with seeking health services, we directly asked participants “When you have sought professional help to stop consuming, what difficulties have you encountered? (Select all that apply)” and presented a list with different options. The participant could choose all that applied in their case and add another if necessary.

All the questions that measured the global variables and their answer options, except for depression, were obtained with the help of three experts in the field of substance use. A template with the questions and instructions was sent to each expert explaining the context of the investigation, the context of the research, the dimension, and the indicator that each item or group of items measures, and the use that the results will have. The experts evaluated the template individually and made comments and suggestions. Finally, their responses were analyzed, and a consensus was obtained.

The BS was developed in Google Forms and applied face to face through electronic tablets. The BS included open-ended and multiple-choice questions. None of the questions recorded the participant’s identification data, so their information remained anonymous.

### 2.3. Procedure

The recruitment of the BS was carried out at the facilities of each residential center, by the research team, with the support of final semester Psychology students from the Autonomous University of Aguascalientes. Contact with the residential centers was made through the ISSEA and once it was established, the team visited each center to administer the BS in person.

The application began by verbally explaining the research objectives to the participants and asking for their consent to answer the BS. The BS was administered from November 18th, 2021, to August 23rd, 2022.

### 2.4. Ethical considerations

At the beginning of each survey administration, the participants were informed about the objectives of the research and asked for their consent to participate in the study. They were also informed that their data would be used for research purposes only and that only the research team would have access to it. In addition, it was clarified that there would be no punishment or benefit in the residential center consequently for their participation.

Information security was carried out following the ethical standards of the Autonomous University of Aguascalientes, which guarantees that the participants’ data will only be used for research purposes by the team that conducted the study. The members of the Institutional Committee of Bioethics approved the ethics and scientific aspects of the protocol used in this study in the CIB-UAA-32 letter.

### 2.5. Data analysis

The data analysis included the global variables of consumption history and barriers to the use of services. First, we converted ordinal variables (Annual and Monthly consumption, Dangerous perception of meth use, Longest period without meth use, Attempts to stop consumption, and Desire to stop consuming) to numeric for statistical analysis. To do this, we used the Map ordinal values to numbers method, which consists of creating a map to reflect each value to a number (Johansson, 2019). For example, for the annual and monthly consumption variables, we converted the answer values as the followings: I did not consume it = 0, 1 to 3 times = 1, 4 to 11 times = 2, and More than 12 times = 3 (see section “2.2. Instrument” for a complete description). Then, to determine the prevalence of each variable, an exploratory analysis was performed with descriptive statistics, where the frequency and percentage were calculated. To determine the relationship between consumption and barriers a correlation test was performed using the non-parametric Spearman’s correlation test, which can be used to determine if there is a relationship between two or more variables when they do not follow a normal distribution. Distribution was tested using the Shapiro-Wilk test for Multivariate Normality. Once the continuous variables most strongly associated with barriers were identified, a clustering analysis was performed using the K-means (KM) algorithm on these variables. KM was used to identify the frequency of initially unknown clusters, since this algorithm is useful when there is a large amount of data, but its structure is unknown (Johansson, 2019). In the case described here, the algorithm was used to find patterns in the consumption and barriers of the participants. The algorithm starts with a given number of clusters (k), then randomly initializes the centroids (with an x, y, z coordinate system where each value corresponds to a variable) of each k and assigns each data to the closest centroid (Hearty, 2016). Once all data is assigned to a centroid, the centroid is recalculated, and the previous step is repeated (Hearty, 2016). The algorithm ends when there is no change in the position of the centroids, and each data has a centroid assigned (Hearty, 2016).

To determine the number of k, we use the technique known as The Elbow Method, which consists of comparing the average distance between the data points and their centroid (Bholowalia and Kumar, 2014). Since the value of the average decreases as we increase k, it is recommended to use the average distance to the centroid as a function of k and find the “elbow point,” where the rate of decrease becomes steeper (Bholowalia and Kumar, 2014).

The KM algorithm was implemented using Python 3.9 and sklearn.cluster module from the scikit-learn library. To perform KM with this library, it is necessary first to train it with the dataset using the *fit* method, and then obtain the result of the clustering by calling the *predict* method (Johansson, 2019). To obtain the clustering results, we used the *fit* and *predict* method. Because the KM algorithm is sensitive to outliers (Hearty, 2016), we first implemented this algorithm with and without outliers to define the data set on which the analysis would be performed. We removed outliers based on the interquartile range (which does not require the data to be normally distributed). Once we identified the data set, we used the *fit* and *predict* methods on the data set. Since KM is an unsupervised machine learning algorithm, where there is no labeled data as ground truth to compare the output of the clustering to the true labels to evaluate its performance, it is possible to use the entire data set, without splitting the data set into a training sample and test sample (Hearty, 2016). Then, we use the Silhouette score to measure the performance of the KM algorithm. This is a good measure when there is unlabeled data and can be used to evaluate how well-defined the clusters are (Hearty, 2016). This score is bounded between −1 and 1, where values close to −1 indicates incorrect clustering, close to 1 very dense clustering, and around 0 indicate overlapping clusters (Hearty, 2016).

Finally, we compared each variable between the cluster resulting from the KM analysis, using Mann–Whitney *U*-test and the Shapiro-Wilk test for distributions. The statistics were carried out with the JASP statistical analysis software, and a significant level of α = 0.05 was used.

## 3. Results

Of the 865 participants, 84.9% were men (*n* = 734), 14.3% were women (*n* = 124), and 0.8% (*n* = 7) preferred not to indicate their gender; 91.9% were dwellers of the Aguascalientes state, of which 58.2% lived in the capital city and the rest in other municipalities. The average age of the participants was 26.8 years.

It was found that, on average, the participants reported 2.12 barriers, and the most common were: not considering treatment services useful for them (41.6%), not considering it important to participate in their treatment (35%), and not finding time to attend a consultation (29.8%). Table 1 shows the results of the barriers reported by the participants.

**TABLE 1:** Frequency and percentage per barrier.

Barrier	F	%
You do not consider that these types of services are useful for you	360	41.6
It is not important for you to attend treatment	303	35
You couldn’t find the time to attend	258	29.8
Cost of consultations	208	24
Location of the place where they serve	196	22.7
Transport	165	19.1
Time for the first date	123	14.2
Waiting time between one appointment and another	112	12.9
Therapist attitudes	112	12.9
Others	43	4.9

Regarding the meth use variables, it was found that on average, participants reported using 4.8 substances at some point in their lives and the most common were tobacco (87.5%), alcohol (84.7%), and cannabis (82.3%); the average age of onset of meth use was 19.8 years; the average length of time they had used meth was 7.08 years. Regarding meth use before intake at the residential center within the previous year, 80.8% (*n* = 699) of people reported more than 12 consumption episodes, 9.4% (*n* = 81) reported between three to 11 episodes, and 4% (*n* = 35) one or two episodes. Some participants did not use meth before treatment 5.8% (*n* = 50) for 12 months. Respect the previous month, 55.1% (*n* = 477) participants reported more than 12 episodes, 17.2% (*n* = 149) between three to 11 episodes, 10.3% (*n* = 89) one or two episodes, and 17.4% (*n* = 150) did not use meth before treatment. Those people mentioned that legal problems brought them to the treatment center, that they have consumed meth in the past and referred to it as their drug of impact. A total of 42% of the participants perceived their use as very dangerous, 36% as dangerous and 22% as not dangerous.

It was found that the highest number of times without meth use was: 47% abstained between 2 and 6 months, 20.6% between 8 days and 1 month, 24.11% between 1 and 7 days, and 8.3% reported continuous use of meth. A total of 90.4% of respondents reported trying to quit meth at some point in their lives, and 97.1% wanted to quit at the time of the survey. Finally, participants reported an average of 5.9 problems associated with consumption, the most frequent were: weight loss (94.9%), sleep problems (80.4%), anxiety (75.3%), and violent behavior (64.7%). Table 2 shows these results.

**TABLE 2:** Frequency and percentage per associated problem.

Associated problem	F	%
Weight loss	821	94.9
Sleeping difficulties	696	80.5
Anxiety	652	75.4
Violent behavior	560	64.7
Confusion	473	54.7
Dental problems	450	52
Hallucinations	449	51.9
Paranoia	416	48.1
Memory loss	373	43.1
Intense itching	263	30.4
Others	40	4.6

The verification of the assumption of normality with the Shapiro-Wilk test for Multivariate normality was significant (*W* = 0.921, *p* < 0.001), suggesting that the data is not normally distributed. The Spearman’s correlation analysis indicated that the use variables that showed a statistically significant relationship, although weak, with the number of barriers reported by participants were: the age of onset of meth use [*r*(863) = −0.83, *p* = 0.015], dangerous perception of meth use [*r*(863) = 0.103, *p* = 0.002], attempts to quit [*r*(863) = 0.083, *p* = 0.016], and the number of problems associated with use [*r*(863) = 0.187, *p* < 0.001]. These results are shown in Table 3.

**TABLE 3:** Correlation table between consumption variables and the number of barriers.

		Spearman	
		rho	*p*
No. barriers	Total drugs used	0.043	0.208
	Meth use onset age	−0.082	0.015
	Time consuming (years)	0.017	0.625
	Annual consumption	0.008	0.805
	Monthly consumption	0.031	0.363
	Dangerous perception of meth use	0.103	0.002
	Longest period without meth use	0.012	0.721
	Attempts to stop consumption	0.082	0.016
	Problems associated with consumption	0.187	<0.001

**FIGURE 1:**
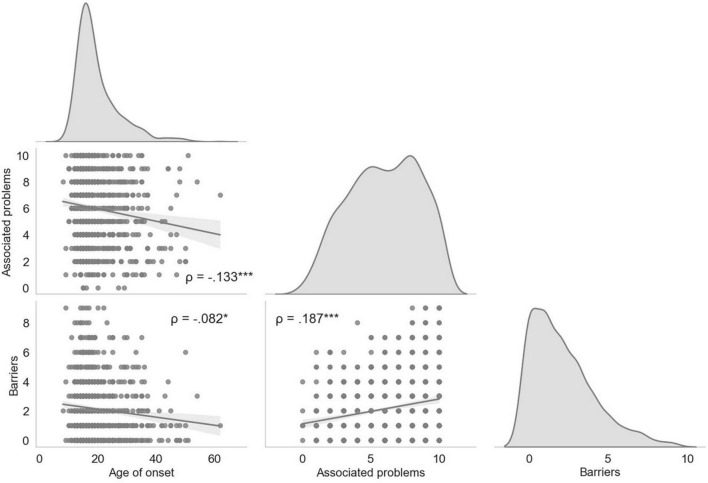
Correlation matrix between the main consumption variables and the number of barriers presented. The correlation matrix shows the density plots for each variable, and the scatter plots the distribution of each participant by the correlations between the variables. Each point represents the results of the participants. *p = 0.015; ***p < 0.001.

To perform the cluster analysis with KM, the continuous variables that showed a statistically significant relationship in the Spearman’s correlation analysis, were used since this algorithm works better with this type of variables. These variables were: the age of onset of meth use and the number of problems associated with consumption as is shown in Figure 1.

**FIGURE 2:**
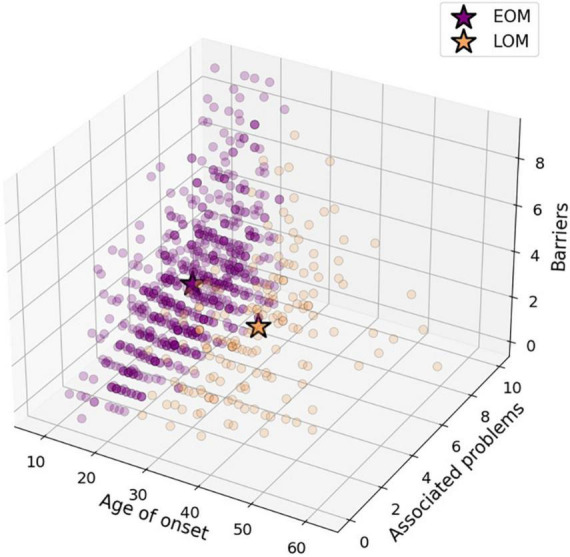
Scatter plot of patients distributed by the main consumption variables and the number of barriers presented.

The results of the elbow point analysis showed that the optimal number of k was two, since that was where the smallest difference between the mean distance between the data points was found. This was confirmed with the silhouette analysis, where we found a score of 0.35 for *k* = 40.44 for *k* = 3, and the highest and closest to 1, which was 0.57 for *k* = 2. Since the results of the KM algorithm with and without outliers pointed in the same direction (the number of outliers in each cluster were proportional), and because we consider that the possible outliers could have been produced by a natural variation that is part of the population we are studying, we use the complete data set, without removing the outliers. Figure 2 shows each of the clusters that the KM algorithm found, represented by each color. As can be seen, the algorithm found two patterns or clusters. One cluster is formed by patients who started consuming meth at a young age, who reported more problems associated with use and more barriers in seeking services (defined as Early Onset of Meth use group or EOM to shorten; *n* = 691; purple dots in Figure 1), while the other cluster is formed by patients who started consuming at an older age, who reported fewer associated problems and fewer barriers (defined as Late Onset of Meth use group or LOM; in orange). The EOM group consisted of 588 men and 103 women, while in the LOM group there were 146 men and 28 women.

The average age of onset of meth use in the EOM group was 16.68 years, with a range between 8 and 24 years. Their current average age is 23.93 years. The age of onset for the LOM group was 32.16 years, with a range between 25 and 62 years. Their current average age is 38.54 years. The average time-consuming meth was 7.25 years for the EOM group and 6.38 years for the LOM group. The lifetime meth consumption ratio in the EOM group was 30%, while in the LOM group it was 16%. The average number of problems associated with meth consumption was 6.1 for the EOM group and they reported an average of 2.21 barriers. For the LOM group, these results were 5.37 and 1.77, respectively.

**FIGURE 3:**
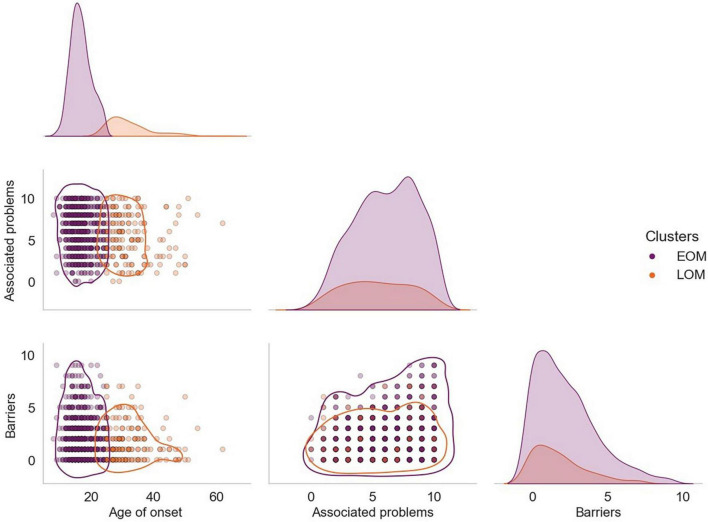
Correlation matrix between the main consumption variables and the number of barriers presented by group. The correlation matrix shows density plots for each variable and scatter plots for the distribution of each participant by the correlations between variables. Each point represents the results of the participants. The purple-colored points correspond to the group EOM, while the orange-colored points correspond to the group LOM.

In Figure 3, the distribution of the variables age of onset of meth use, number of associated problems, and number of barriers for both groups can be observed.

Finally, Table 4 shows the comparison of all the variables using the Mann–Whitney *U*-test for the groups in order to observe the differences between the group EOM and the LOM group. The verification of the assumption of normality with the Shapiro-Wilk test was significant in all cases (*p* < 0.001), suggesting a deviation from normally. Significant differences were obtained in total consumption (*U* = 67102, *p* = 0.016), where the EOM group reported consuming more substances (*M* = 4.95, *SD* = 1.68) than the LOM group (*M* = 4.59, *SD* = 1.78), in problems associated with consumption (*U* = 69415, *p* = 0.001), where those who started EOM reported more problems (*M* = 6.1, *SD* = 2.53 vs. *M* = 5.37, *SD* = 2.71). Similarly, the EOM group reported more barriers (*M* = 2.21, *SD* = 2.04) and time consuming (*M* = 7.25, *SD* = 5.44) on average than the LOM group (*M* = 1.77, *SD* = 1.78; *M* = 6.38, *SD* = 4.38; respectively) and this difference was statistically significant (*U* = 67395, *p* = 0.012; *U* = 66561, *p* = 0.028; respectively).

**TABLE 4:** Comparison between all variables by group.

Variable	EOM				LOM				*p*	U	Effect size
	Mean	Median	IQR	SD	Mean	Median	IQR	SD			
Total drugs used	4.94	5	2	1.68	4.59	5	3	1.78	0.016	67102	0.116
Meth use onset age	16.68	16	5	3.26	32.16	30	8	7.06	<0.001	0	−1
Current age	23.94	23	7	6.41	38.54	38	8.75	7.48	<0.001	7152	−0.881
Time consuming (years)	7.25	6	5	5.44	6.38	5	6	4.38	0.028	66561	0.107
Annual consumption	2.66	3	0	0.8	2.61	3	0	0.82	0.269	62354	0.037
Monthly consumption	2.12	3	0	1.15	2	2.5	0	1.16	0.15	63960	0.064
Dangerous perception of meth use	1.12	1	1	0.74	1.05	1	2	0.79	0.261	63209	0.051
Longest period without meth use	0.97	1	2	1.03	0.85	0.5	2	0.98	0.196	63679	0.059
Attempts to stop consumption	0.9	1	0	0.3	0.9	1	0	0.29	0.842	59816	−0.005
Problems associated with consumption	6.1	6	4	2.53	5.37	5	5	2.71	0.001	69415	0.155
Barriers	2.21	2	2	2.04	1.77	1	3	1.78	0.012	67395	0.121

The annual and monthly consumption where: I didn’t = 0, 1 to 3 times = 1, 4 to 11 times = 2, and More than 12 times = 3, where the average close to 3 indicates that most participants had more than 12 episodes of use. The Dangerous perception of meth use was averaged with: It is not dangerous = 0, It is dangerous = 1, It is very dangerous = 2, where the average close to 1 indicates that most participants consider their consumption dangerous. The Greatest number of days without consumption was averaged with: From 2 to 6 months = 0, From 8 days to 1 month = 1, From 1 to 7 days = 2, 0 days = 3, the average close to 1 indicates that most participants had between 8 days and 1 month of abstinence. Lastly, attempts to stop consumption were obtained with: No = 0, Yes = 1. The average close to 1 indicates that most of the participants have tried to stop consumption at some time in their lives.

## 4. Discussion

The present study aimed to identify the main barriers to seeking treatment for addiction among patients receiving residential treatment in the state of Aguascalientes, to determine the relationship between these barriers and meth use, and to identify profiles of users based on this relationship. It was found that the participants reported an average of 2.12 barriers, with the most common being that they did not think this service was for them, that they did not value attending appointments, and that they did not have the time to attend appointments. An important fact of these findings is that the participants mentioned these barriers while receiving residential treatment. This could imply that they have not found the benefit of the treatment and still consider that these types of services are not useful.

The main barrier found was that users did not perceive treatment services to be useful, which is like a study that identified a low perceived need for treatment as a specific barrier to retention in substance use treatment (Zemore et al., 2021). It should be noted that one of the studies focuses primarily on barriers to substance use treatment retention rather than barriers to meth treatment access (Cumming et al., 2016).

In another study (Wallace et al., 2009), there was a lack of recognition of the benefits of treatment among meth users, which is in line with our findings. However, in that study, this barrier was only present among users who had not received any form of support compared to those who had received some form of treatment, as they perceived the benefits of having undergone treatment.

Another barrier reported by users is that seeking treatment is not seen as important, which is consistent with the specialized literature (Wallace et al., 2009), where the main barrier to seeking treatment is a lack of perceived need and motivation to seek support. This may be related to stigma, which is a barrier that affects people with these types of disorders by having incorrect assumptions or perceptions about care services (Zwick et al., 2020). There is a need to raise awareness and disseminate the effectiveness of treatments specifically for meth.

Regarding meth use, the main findings were that participants reported using an average of 4.8 substances at some point in their lives, with an average age of onset of use of 19.8 years and reported an average of 5.9 problems associated with their drug use.

In this study, we identified the difficulty of finding time to attend appointments as a barrier, compared to another study conducted by Guerrero et al. (2014) where this type of structural barrier was identified more in that treatment centers that are located far away, and users mentioned difficulties in accessing the service. In the study conducted, this barrier is related to the difficulty of finding the place where the treatment is provided and a form of transportation, which hinders access to the treatments offered.

Regarding the identification of user profiles, we found two profiles or clusters of participants, one formed by participants that started consumption at a young age, and have more problems associated with meth use and more barriers in seeking services, and another profile formed by individuals who start at an older age, reported fewer associated problems and barriers. This result arises from the analysis of the KM machine learning algorithm. This result may have important implications for the design and implementation of meth uses prevention and treatment programs.

Another result is that statistically significant differences were found using the Mann–Whitney *U*-test, between these profiles or groups in the number of substances consumed, time consuming, number of barriers and number of problems associated with consumption, where the EOM group consumed more substances and has more time (in year) consuming, presented more associated problems, and more barriers than those who started older. The results of the study can be explained overall by the fact that participants in the early onset meth use (EOM) group began using meth earlier than participants in the late-onset meth use (LOM) group. This allowed EOM participants to have a greater proportion of time-consuming meth (30%), while LOM participants did not. In other words, the age of onset can have the effect of increasing the percentage of an individual’s life that is lost to meth use. This lifetime meth consumption ratio could explain why EOM users, even at a much younger age, still differ from LOM users in terms of the perceived number of barriers to seeking treatment and the reported number of problems associated with meth use. This suggests that it may not be the age at which meth use began that is the determining factor, but rather the amount of time that an individual spends using meth. More research is needed to understand the long-term effects of meth use on different populations.

The early onset meth use group reported more substance use and more time using than the late onset meth use group. This may be due to multiple factors like individual factors, social factors, and drug availability (Tena-Suck et al., 2018). This means that could be not since they started consuming meth in adolescence, but rather that they proportionally spent more time of their lives using drugs. The specialized literature indicates that adolescents are at a higher risk of consuming substances because they are influenced by a complex interaction between various aspects of their development, such as greater impulsivity and a tendency to show reckless behaviors (Tena-Suck et al., 2018). However, it is suggested to consider that the increasing use of meth has increased in people in general, but mainly in those who consume opioids (Bach et al., 2020), which means that consumption corresponds to more than one substance in new generations of young people, which is becoming a particularly difficult problem. Therefore, the high consumption of different drugs at an early age suggests a focus on care and, above all, prevention programs for the young population.

Another contribution of the study is the association between the younger age of onset of meth use and a higher number of associated problems and barriers. This aspect has not been identified in other studies that have focused on attitudinal and structural barriers to help-seeking in substance use populations based on gender and race or ethnicity (Otiniano and Grella, 2017). Age could predict negative outcomes associated with meth use, and early onset or re-initiation of use is a significant factor in the development of meth use and help seeking. Therefore, the age of onset becomes an important indicator in the development and help-seeking for meth use.

The data offer a particular view, given the sample, but allow us to reflect on the fact that the age of initiation of meth use is related to a greater number of barriers to access to treatment, which allows future research to support efforts to deepen the knowledge of the type of barriers according to different age groups. Another aspect that could be implemented with the results is to sensitize users about the benefits of entering treatment, informing them about the effects of substance use and the user’s own responsibilities when receiving treatment.

## 5. Conclusion

These results suggest that the perceived usefulness of treatment, motivation to address drug use, and availability of time to attend consultations are the main barriers that patients in Aguascalientes face when seeking treatment for meth use. It is important to note that these barriers are related to both psychological and practical factors, which highlights the need to address not only the clinical aspects of treatment but also the individual and contextual needs of the patients. In addition, these findings may be useful in the design of interventions to improve the accessibility and quality of treatment services for meth users in Aguascalientes and other regions with similar characteristics.

The barriers identified in this study are consistent with previous literature on difficulties in accessing treatment services and a lack of perception of the benefits of treatment. However, it is relevant to consider structural barriers, such as location and accessibility to treatment services, as they may also limit people’s ability to seek care. All the above allows future research to analyze the implications of barriers to the design and implementation of programs and treatments for meth use, one of which could be the implementation of telemedicine or online care to favor the understanding of treatment and the benefits that would be obtained, as an antecedent to the intervention of residential treatment. In summary, the results suggest that there are different profiles of meth use among study participants, and that these profiles are associated with different levels of problems associated with meth use and barriers. This may have important implications for the design and implementation of meth uses prevention and treatment programs.

### 5.1. Study limitations

An important limitation of the present study was that the barriers were addressed using closed-ended questions during the administration of the brief survey. Although our results are consistent with the specialized literature, we believe that further studies are needed to address these barriers and the variables related to them. A qualitative approach with in-depth interviews could shed light on how the age of onset affects patients with more barriers and associated problems. We also consider that it is important to address how addiction and treatment services can be improved from the user’s perspective to overcome these barriers.

We believe that this study has a high degree of reporting bias, since the survey we used was applied to people who were receiving treatment, without considering people who do not have access to these services. This bias may mean that the barriers are different for those people who may have little chance of entering these institutes.

Another important limitation is that the study was conducted in a single region, Aguascalientes, which limits the generalizability of the results to other geographic and cultural areas. Further studies in different regions with larger samples are needed to obtain a more comprehensive understanding of the barriers faced by meth users when seeking treatment in Mexico. In addition, the study sample was recruited through a network of contacts, which may have biased the selection of participants and limited the representativeness of the sample.

Finally, it is important to note that the study focused exclusively on patient-perceived barriers and did not address structural barriers such as lack of access to treatment services or lack of funding for mental health services. Addressing these barriers is necessary to improve the accessibility and quality of care for meth users.

## Data availability statement

The raw data supporting the conclusions of this article will be made available by the authors, without undue reservation.

## Ethics statement

The studies involving humans were approved by the Institutional Committee of Bioethics. The studies were conducted in accordance with the local legislation and institutional requirements. Written informed consent for participation in this study was provided by the participants’ legal guardians/next of kin.

## Author contributions

KM and YO contributed substantially to the conception, design, acquisition, analysis, and interpretation of data for the work. LR contributed by revising the work critically for important intellectual content. MP contributes with drafting the work. All authors contributed to the article and approved the submitted version.

## References

[B1] Alexander A. C. Obong’o C. O. Chavan P. P. Dillon P. J. Kedia S. K. (2018). Addicted to the ‘life of methamphetamine’: Perceived barriers to sustained methamphetamine recovery. *Drugs Educ. Prevent. Policy* 25 241–247. 10.1080/09687637.2017.1282423

[B2] Auerbach R. P. Mortier P. Bruffaerts R. Alonso J. Benjet C. Cuijpers P. (2018). WHO World Mental Health surveys international college student project: Prevalence and distribution of mental disorders. *J. Abnorm. Psychol.* 127 623–638. 10.1037/abn0000362 30211576 PMC6193834

[B3] Bach P. Hayashi K. Milloy M. J. Nosova E. Kerr T. Wood E. (2020). Characterising the increasing prevalence of crystal methamphetamine use in Vancouver. Canada, from 2006-2017: A gender-based analysis. *Drug Alcohol Rev.* 39 932–940. 10.1111/dar.13126 32666650 PMC7683370

[B4] Benjet C. Wittenborn A. Gutierrez-García R. A. Albor Y. C. Contreras E. V. Hernández S. C. (2020). Treatment delivery preferences associated with type of mental disorder and perceived treatment barriers among Mexican University Students. *J. Adolesc. Health* 67 232–238. 10.1016/j.jadohealth.2020.01.025 32169528

[B5] Bholowalia P. Kumar A. (2014). EBK-means: A clustering technique based on elbow method and k-means in WSN. *Int. J. Compute. Appl.* 105 17–24.

[B6] Borges G. Medina-Mora M. E. Wang P. S. Lara C. Berglund P. Walters E. (2006). Treatment and adequacy of treatment of mental disorders among respondents to the Mexico National Comorbidity Survey. *Am. J. Psychiatry* 163 1371–1378. 10.1176/ajp.2006.163.8.1371 16877649

[B7] Borges G. Wang P. S. Medina-Mora M. E. Lara C. Chiu W. T. (2007). Delay of first treatment of mental and substance use disorders in Mexico. *Am. J. Public Health* 97 1638–1643. 10.2105/AJPH.2006.090985 17666703 PMC1963297

[B8] Clifford B. Van Gordon K. Magee F. Malone V. Siefried K. J. Graham D. (2023). “There’s a big tag on my head”: Exploring barriers to treatment seeking with women who use methamphetamine in Sydney. Australia. *BMC Health Serv. Res.* 23:162. 10.1186/s12913-023-09125-z 36793060 PMC9933255

[B9] Comisión Nacional contra las Adicciones [CONADIC] (2020). *Informes sobre la demanda de tratamiento por consumo de sustancias psicoactivas.* Available online at: http://www.gob.mx/saludconadic/acciones-y-programas/informes-sobre-la-demanda-de-tratamiento-por-consumo-de-sustancias-psicoactivas?state=published (accessed March 2023).

[B10] Crisp A. H. Gelder M. G. Rix S. Meltzer H. I. Rowlands O. J. (2000). Stigmatization of people with mental illnesses. *Br. J. Psychiatry* 177 4–7. 10.1192/bjp.177.1.4 10945080

[B11] Cumming C. Troeung L. Young J. T. Kelty E. Preen D. B. (2016). Barriers to accessing methamphetamine treatment: A systematic review and meta-analysis. *Drug Alcohol Depend.* 168 263–273. 10.1016/j.drugalcdep.2016.10.001 27736680

[B12] Guerrero E. G. Aarons G. A. Grella C. E. Garner B. Cook B. Vega W. A. (2014). Program capacity to eliminate outcome disparities in addiction health services. *Adminis. Policy Ment. Health Ment. Health Serv. Res.* 43 23–35. 10.1007/s10488-014-0617-6 25450596 PMC4452456

[B13] Hearty J. (2016). *Advanced machine learning with Python.* Birmingham: Packt Publishing Ltd.

[B14] Issakidis C. Andrews G. (2002). Service utilisation for anxiety in an Australian community sample. *Soc. Psychiatry Psychiatr. Epidemiol.* 37 153–163. 10.1007/s001270200009 12027241

[B15] Johansson R. (2019). *Numerical Python.* New York, NY: Apress.

[B16] Martínez K. I. Ojeda Y. L. Hernández J. Contreras-Pérez M. E. (2023). Depression and suicidal behavior comorbidity in patients admitted to substance-use residential treatment in aguascalientes. Mexico. *J. Evidence Based Soc. Work* 20 508–519. 10.1080/26408066.2023.2172368 37330687

[B17] National Institute on Drug Abuse [NIDA] (2017). *Health consequences of drug misuse.* Bethesda, MD: National Institute on Drug Abuse.

[B18] National Institute on Drug Abuse [NIDA] (2022). *What is methamphetamine?* Available online at: https://nida.nih.gov/publications/research-reports/methamphetamine/what-methamphetamine (accessed March 2023).

[B19] Otiniano A. D. O. Grella C. E. (2017). Influence of gender and race/ethnicity on perceived barriers to help-seeking for alcohol or drug problems. *J. Subst. Abuse Treat.* 75 54–61. 10.1016/j.jsat.2016.12.013 28237055 PMC5329903

[B20] Quinn B. Stoové M. Papanastasiou C. Dietze P. (2013). An exploration of self-perceived non-problematic use as a barrier to professional support for methamphetamine users. *Int. J. Drug Policy* 24 619–623. 10.1016/j.drugpo.2013.05.015 23867050

[B21] Saldivia S. Vicente B. Kohn R. Rioseco P. Torres S. (2004). Use of Mental Health Services in Chile. *Psychiatr. Serv.* 55 71–76. 10.1176/appi.ps.55.1.71 14699204

[B22] Sareen J. Jagdeo A. Cox B. J. Clara I. ten Have M. Belik S.-L. (2007). Perceived barriers to Mental Health Service Utilization in the United States, Ontario, and the Netherlands. *Psychiatr. Serv.* 58 357–364. 10.1176/ps.2007.58.3.357 17325109

[B23] Sistema de Vigilancia Epidemiológica de las Adicciones [SISVEA] (2020). *Informe SISVEA 2020.* Available online at: https://www.gob.mx/cms/uploads/attachment/file/746477/informe_sisvea_2020.PDF (accessed March 2023).

[B24] Tena-Suck A. Castro-Martínez G. Marín-Navarrete R. Gómez-Romero P. Fuente-Martín A. Gómez-Martínez R. (2018). Consumo de sustancias en adolescentes: Consideraciones para la práctica médica. *Med. Int. México* 34:264277. 10.24245/mim.v34i2.1595

[B25] United Nations Office on Drugs and Crime [UNODC] (2022). *World drug report 2022.* Avaliable online at: https://www.unodc.org/unodc/en/data-and-analysis/wdr-2022_booklet-4.html (accessed March 2023).

[B26] Wallace C. Galloway T. Mcketin R. Kelly E. Leary J. (2009). Methamphetamine use, dependence and treatment access in rural and regional North Coast of New South Wales, Australia. *Drug Alcohol Rev.* 28 592–599. 10.1111/j.1465-3362.2008.00016.x 19930011

[B27] Zemore S. E. Ware O. D. Gilbert P. A. Pinedo M. (2021). Barriers to retention in substance use treatment: Validation of a new, theory-based scale. *J. Subst. Abuse Treat.* 131:108422. 10.1016/j.jsat.2021.108422 34098296 PMC8528875

[B28] Zwick J. Appleseth H. Arndt S. (2020). Stigma: How it affects the substance use disorder patient. *Subst. Abuse Treat. Preventi. Policy* 15:50. 10.1186/s13011-020-00288-0 32718328 PMC7385978

